# The atherogenic dyslipidemia ratio [log(TG)/HDL-C] is associated with residual vascular risk, beta-cell function loss and microangiopathy in type 2 diabetes females

**DOI:** 10.1186/1476-511X-11-132

**Published:** 2012-10-09

**Authors:** Michel P Hermans, Sylvie A Ahn, Michel F Rousseau

**Affiliations:** 1 Division of Endocrinology and Nutrition, Université catholique de Louvain, Brussels, Belgium; 2 Division of Cardiology, Université catholique de Louvain, Brussels, Belgium

**Keywords:** HDL-C, Triglycerides, Cardiovascular risk, Microangiopathy, β-cell function, Hyperbolic product, Gender, Diabetes

## Abstract

**Background:**

Atherogenic dyslipidemia (AD), defined as low HDL-C plus elevated triglycerides (TG), comorbid to T2DM, increases cardiometabolic risk for CAD even when LDL-C is at target. In T2DM males, AD was shown to correlate with β-cell function loss, yet it is not established whether this applies across gender.

**Aim:**

To establish the prevalence and severity of AD in T2DM females, and to determine how it relates to cardiometabolic phenotype, glucose homeostasis, micro- and macrovascular complications, and 10-year absolute CV risk (*UKPDS* Risk Engine).

**Methods:**

340 T2DM females were ranked according to quintiles (Q) of the continuous variable *log*(TG)/HDL-C, with AD prevalence defined as HDL-C <50 mg.dL^-1^ plus TG ≥150 mg.dL^-1^, and β-cell function assessed with HOMA.

**Results:**

AD prevalence was 35%; mean HDL-C and TG were 52 (15) and 160 (105) mg.dL^-1^. AD was significantly related to central fat, metabolic syndrome, sedentarity and skeletal sarcopenia, as well as to _hs_CRP, fibrinogen, uric acid, cystatin-C, Big ET-1, and 10-year *UKPDS* CV risk. AD correlated stepwise with lower β-cell function and hyperbolic product, and with accelerated loss of residual insulin secretion, higher HbA_1c_ and prevalent microangiopathy.

**Conclusions:**

*log*(TG)/HDL-C is a simple means to grade AD and residual macrovascular risk in T2DM females. This ratio associates with major non-LDL cardiometabolic variables and ranks predicted CAD risk. In addition, *log*(TG)/HDL-C identifies worsening glucose homeostasis, poorer glycemic control, and prevalent microangiopathy.

## Introduction

Current guidelines recommend intensive lowering of low-density lipoprotein cholesterol (LDL-C) in type 2 diabetes mellitus (T2DM) patients [1-5]. The common form of T2DM, associated with insulin resistance (IR) and the metabolic syndrome (MetS) is characterized by a singular, non-LDL dyslipidemia, defined as atherogenic dyslipidemia (AD). The hallmark of AD is decreased levels of high-density lipoprotein cholesterol (HDL-C) together with raised triglycerides (TG). AD substantially contributes to residual vascular risk (RVR), even when LDL-C is at or below targets, both in diabetic and nondiabetic populations. The presence and/or severity of AD can be established either from the *combined* occurrence of high TG levels and low HDL-C or from the *ratio* of fasting TG to fasting HDL-C, with prior *log* transformation of TG levels allowing to compute a broad range of TG values [2-11]. Whereas screening for AD provides clinically relevant information for assessing RVR, it is rarely performed due to lack of agreement or consensual cut-offs to define/grade AD from baseline HDL-C and TG levels prior to lipid-lowering therapies (LLD).

We previously reported that *log*(TG)/HDL-C is a useful measure of increased cardiometabolic risk in T2DM males, in whom the ratio also relates to poorer metabolic control and β-cell function loss [7]. In diabetic females, there exists a reversed dichotomy in risk factors for CAD vis-à-vis nondiabetic women, the former no longer benefiting from female gender’s risk protection. Thus, diabetic women have more severe dyslipidemia, more prevalent obesity and abdominal fat, higher subclinical inflammatory markers, more severe/prevalent hypertension or MetS. Worryingly, diabetic women are less often at targets for major modifiable micro- and macrovascular risk factors, leaving them exposed to higher RVR than males [12-15]. The aim of the present study was (*i*) to establish and grade AD prevalence in a cohort of T2DM females using the continuous variable *log*(TG)/HDL-C; (*ii*) to determine whether this ratio relates to their cardiometabolic phenotype or glucose homeostasis determinants; and (*iii*) to assess the association between AD and micro- or macrovascular complications, and its impact on absolute 10-year CV risk from the *United Kingdom Prospective Diabetes Study* (UKPDS) calculator [16].

## Methods

This cross-sectional study included 340 female T2DM outpatients, attending the Diabetes Clinic between January 2008 and June 2011. Patients were divided according to quintiles (Q) of baseline, pre-LLD *log*(TG)/HDL-C values. The following variables were recorded: age, ethnicity, attained educational level (dichotomised as low *vs.* high, based on higher achieved education degree [no education, primary or secondary *vs.* higher education and university]), known diabetes duration, family history (CVD, diabetes mellitus), self-reported leisure-time (LT) weekly exercise duration, and daily-time spent watching screen(s) (television, computers and/or visual numeric media), as surrogate for LT sedentarity [7]. The presence of a MetS (score ≥3/5) was defined according to the joint harmonized *IDF/NHLBI/AHA/WHF/IAS/IASO* criteria [17,18].

Pre-LLD values were retrieved from patients’ records, and used to assess AD prevalence in LLD-treated patients; otherwise, in non-LLD-treated patients, current lipids and AD prevalence were obtained from last available lipid values from the same specimen. In LLD-treated patients, current lipids values were those from last available, post-LLD lipid values. AD *prevalence* was defined as the combination of MetS criteria for low HDL-C in females (<50 mg.dL^-1^) and high fasting TG (≥150 mg.dL^-1^ for both genders) at baseline. AD *severity* was assessed from *log*(fasting TG)/fasting HDL-C. *Normal values* for *log*(TG)/HDL-C, from 44 healthy white Caucasian females: 0.036 (*mean*); 0.034 (*median*); 0.014 (*SD*); 0.014 (*min.*); 0.094 (*max.*); 0.025 (*perc.* 25) and 0.042 (*perc.* 75) [7].

Patients were measured for body weight, height, body mass index (BMI), relative and total body fat (BodyFat Analyzer, Omron BF 500), waist circumference and conicity index. Ultrasonographic evidence for fatty liver was considered in the presence of hyperreflectivity in the absence of etiological factors associated with liver steatosis, including excess ethanol intake [19-25]. Computer-based *Homeostasis Model Assessment* (HOMA) of insulin sensitivity and β-cell function was previously detailed ( http://www.dtu.ox.ac.uk). Values of HOMA B (%) were plotted as a function of HOMA S (%), defining a hyperbolic product area (β×S) [unit: %^2^; normal value: 100%, corresponding to 10^4^ %^2^, which represents the true, underlying β-cell function. (BxS) loss over a subject’s lifetime span was obtained by dividing 100-(BxS) by subjects’ age at the time of HOMA-modeling, providing an estimate of annual (BxS) loss rate (%.year^-1^) [26-29].

Current use of oral antidiabetic drugs, insulin, blood pressure (BP)-lowering drugs, aspirin, and LLD(s) was recorded. Hypertension was defined as systolic BP ≥140 mmHg and/or diastolic BP ≥90 mmHg, or current treatment with BP-lowering drug(s). Coronary artery disease (CAD) was inferred from medical history (myocardial infarction, angioplasty, stenting, revascularization surgery and/or significant coronary stenosis confirmed by angiography) and systematic review of procedures, screening (exercise testing, echocardiography) or subclinical disease imaging data in the patient’s records. Stroke was defined according to *UK Prospective Diabetes Study* (UKPDS) criteria: any neurological deficit ≥1 month, without distinction between ischaemic, embolic and haemorrhagic strokes. In patients with multiple CV events, only the first one was considered for prevalence [16]. Peripheral artery disease (PAD) was defined by a medical history of lower-limb(s) claudication and/or clinical or imaging evidence for ischemic diabetic foot, angioplasty, stenting, revascularization surgery and/or significant lower-limb artery stenosis at Doppler ultrasonography and/or angiography. *UKPDS Risk Engine*’s 10-year absolute CV risk for individuals in primary CV prevention was based on the 10 following variables: *known T2DM duration, age, gender, ethnicity, smoking status, chronic atrial fibrillation, HbA*_*1c*_*level, systolic BP, total C* and *HDL-C*[16].

Diabetic retinopathy (DRP) was diagnosed following dilated fundus examination and fluoangiography [30]. The presence of peripheral neuropathy was established from clinical examination (knee and ankle reflexes, Semmes-Weinstein 5.07 monofilament test) and/or 4-limbs electromyography. Normo, micro- and macro-albuminuria were defined as urinary albumin excretion <30 (normo-), 30–299 (microalbuminuria) and ≥300 μg.mg creatinine^-1^ (macro-albuminuria) from first-morning urine sample. Glomerular filtration rate (eGFR) was estimated using the *Modified Diet in Renal Disease* formula [31]. Glycated haemoglobin (HbA_1c_), total cholesterol (C), HDL-C, TG, LDL-C (from Friedewald’s formula), non-HDL-C (by subtracting HDL-C from total C), apolipoproteins (apo) A1 and B_100_, high-sensitivity C-reactive protein (_hs_CRP), Big endothelin-1 [32], uric acid, cystatin C, fibrinogen, leucocytes, total and free testosterone, *sex-hormone-binding globulin*, ferritin, liver enzymes (*aspartate aminotransferase* [AST], *alanine aminotransferase* [ALT], *γ-glutamyl transferase* [γGT]), homocysteine, folic acid and vitamin B_12_ were determined by routine laboratory methods. Each patient gave informed consent, the research was carried out in compliance with the declaration of Helsinki, and the protocol was approved by the local Institutional Review Board (Commission d'Ethique Biomédicale Hospitalo-Facultaire de l'UCL, Faculté de Médecine).

### Statistical methods

Results are presented as means (± 1 standard deviation (SD)) or proportions. The significance of differences between AD quintiles was assessed by one-way analysis of variance for linear trend between means, and by a Chi-squared test for trend for differences in proportions with modified Bonferroni’s adjustment procedure to minimize type 1 error resulting from multiple testing. Results were considered significant or non-significant (NS) for p< or ≥0.05, respectively.

## Results

Patient’s characteristics are described in Table 1. There were 340 T2DM females in the whole cohort, 85% of whom from Caucasian ancestry. AD prevalence was 35%. When patients were divided according to AD quintiles of *log*(TG)/HDL-C, patients in the 2^nd^ quintile (Q II) had a mean *log*(TG)/HDL-C similar to that of control, nondiabetic subjects (see *Methods*), whereas patients from the 3^rd^ quintile (Q III) had a mean *log*(TG)/HDL-C that corresponded to the 75^th^ centile of controls. In the whole T2DM cohort, mean age (1 SD) and diabetes duration were 67 (13) years and 15 (10) years, respectively, without significant trends across quintiles (Qs). Past- or current smoking prevalence was not different between Qs, whereas ethanol intake showed a significant decreasing trend (*p* <0.0001). LT physical activity and screen-watching daily duration were significantly different across Qs, with increased sedentarity and lesser exercise. There was a significant stepwise decrease in the proportion of patients with higher educational level across quintiles, from 44% (QI) to 18% (QV), which nevertheless was above Bonferroni’s adjusted level of significance. Hypertension prevalence was 85% in the whole cohort, with increasing values across Qs, and mean systolo-diastolic BP values 139 (21) / 78 (11) mmHg, without trends across Qs (Table 1).

**Table 1:** Patient’s characteristics

		**all patients**	**Q I**	**Q II**	**Q III**	**Q IV**	**Q V**	**P**
n		340	68	68	68	68	68	
log (TG).HDL-C^-1^		0.045	0.026	0.035	0.042	0.050	0.071	~
age	years	67 (13)	67 (13)	66 (13)	67 (13)	68 (12)	65 (14)	NS
diabetes duration	years	15 (10)	15 (10)	14 (9)	15 (11)	16 (9)	17 (9)	NS
smoking ^§^	%	72-28	75-25	77-23	72-28	68-32	68-32	NS
ethanol	U.week^-1^	5 (11)	9 (14)	8 (13)	5 (11)	3 (6)	2 (3)	0.0001
education	%	71:29	56:44	69:31	73:27	75:25	82:18	0.0007
hypertension	%	85	78	82	85	88	91	0.0181
Hb A_1c_	%	7.87 (1.62)	7.50 (1.82)	7.50 (1.54)	7.94 (1.62)	8.06 (1.50)	8.34 (1.49)	0.0073
Hb A_1c_	mmol.mol^-1^	63 (13)	58 (14)	59 (12)	63 (13)	65 (12)	68 (12)	<0.0001
microangiopathy	%	51	40	45	55	58	60	0.0045
retinopathy	%	30	24	27	40	31	29	NS
peripheral polyneuropathy	%	25	19	16	25	41	28	0.0111
macroangiopathy	%	22	16	18	28	19	26	NS
Epworth score		6 (4)	5 (4)	6 (4)	5 (3)	7 (5)	7 (4)	0.0066
pathological (>9)	%	18	9	17	12	25	25	0.0066
OSAS	%	6	0	1	9	7	12	0.0011

Results are expressed as means (1SD) or proportions (%) §: never vs. former/current; HbA_1c_:glycated haemoglobin; HDL-C: high density lipoprotein cholesterol; OSAS: obstructive sleep apnea/hyponea syndrome; Q: quintile; TG: triglycerides; NS: not significant.

Current glycemic control, inferred from the average of last-available HbA_1c_, was above target (>7.0%) in the whole group, and significantly worsened across Qs, with a mean absolute differences of 0.84% between Q I/Q II and Q V. The proportion of patients at HbA_1c_ target (33% in the whole cohort) also significantly decreased across Qs, from 47% (Q I) to 21% (Q V; *p* <0.0001). Prevalence of any microangiopathy (51% in the whole cohort) significant increased across Qs, from 40% (Q I) to 60% (Q V; *p* 0.0045). The increase in microvascular complications prevalence across Qs remained significant after adjustment for HbA_1c_ (*p* 0.0261 [overall microangiopathy]; *p* 0.0114 [peripheral neuropathy]). When patients were analyzed according to Qs of non-HDL-C, there were no associations between the latter and the frequency of microangiopathy, overall or site-specific. As regards macroangiopathy, there was no trend for higher prevalence across Qs of *log*(TG)/HDL-C: (overall prevalence (22%); CAD (15%); TIA/stroke (6%) and PAD (5%). Epworth’s daytime sleepiness score and sleep apnoea syndrome prevalence significantly increased across Qs, from 5 (4) and 0% (Q I) to 7 (4) and 12% (Q V; *p* 0.0066 and 0.0011, respectively; Table 1).

There were highly-significant trends across Qs for progressively higher values in BMI, waist circumference, fat mass, conicity index, waist-to-height ratio, visceral fat, and fat-free mass index. Mean insulin sensitivity, β-cell function, and the hyperbolic product between them [BxS] were lower than the normal (100%) value in the whole cohort: 57% (HOMA S), 65% (HOMA B), and 28.6% [BxS], respectively. There were stepwise decreases across Qs in HOMA S and [BxS], from 82% (HOMA S) and 35.9% [BxS] (Q I) to 39% (HOMA S) and 22.5% (BxS) (Q V). [BxS] loss rate significantly worsened across Qs: 1.17 %.yr^-1^ (Q I) to 1.52 %.yr^-1^ (Q V). There was a significant increase across Qs in fasting insulinemia: 77 (Q I) to 141 pmol.l^-1^ (Q V; *p*<0.0001). Liver steatosis frequency (64% prevalent in the whole cohort) significantly rose across Qs: 27% (Q I) to 83% (Q V; *p*<0.0001). A MetS phenotype was present in 86% of the entire cohort; there was a highly-significant stepwise rise in both MetS prevalence and score across Qs: 59% and 2.6 (Q I) to 100% and 4.7 (Q V; both *p* <0.0001). The contribution of discrete components to the MetS score for each Q showed significant trends for higher prevalence of hypertension (*p* 0.0181) and enlarged waist circumference (*p* <0.0001; Table 2).

**Table 2:** Cardiometabolic phenotype

		**all patients**	**Q I**	**Q II**	**Q III**	**Q IV**	**Q V**	**P**
n		340	68	68	68	68	68	
body mass index	kg. m^-2^	30.3 (6.6)	26.6 (4.9)	28.9 (6.5)	31.6 (5.8)	31.2 (6.8)	33.2 (6.8)	<0.0001
weight	kg	78.5 (17.4)	69.4 (14.3)	75.9 (17.7)	81.1 (14.5)	81.3 (17.9)	84.9 (18.3)	<0.0001
waist circumference	cm	100 (15)	90 (12)	99 (13)	104 (13)	103 (16)	107 (14)	<0.0001
fat mass	%	41.0 (6.8)	38.5 (7.8)	40.1 (6.7)	42.9 (5.4)	41.2 (6.7)	42.5 (6.2)	0.0006
conicity index	m^2^.kg^-1^	1.32 (0.10)	1.27 (0.10)	1.32 (0.08)	1.34 (0.09)	1.34 (0.10)	1.36 (0.10)	<0.0001
waist. height^-1^		0.62 (0.10)	0.56 (0.08)	0.61 (0.08)	0.65 (0.09)	0.64 (0.11)	0.67 (0.09)	<0.0001
visceral fat	0-30 score	10 (3)	8 (3)	9 (3)	11 (3)	11 (3)	11 (3)	<0.0001
fat free mass index	kg.m^-2^	17.8 (3.4)	17.2 (3.9)	17.3 (3.3)	18.1 (2.3)	17.8 (3.5)	18.8 (3.5)	0.0377
HOMA S	%	57 (42)	82 (52)	67 (45)	46 (34)	45 (26)	39 (30)	<0.0001
HOMA B	%	65 (48)	55 (40)	54 (31)	77 (63)	70 (45)	70 (53)	0.0158
HOMA product [BxS]	%	28.6 (18.5)	35.9 (21.1)	30.6 (20.3)	26.8 (18.0)	25.9 (11.9)	22.5 (17.1)	<0.0001
[BxS] loss rate	%.yr^-1^	1.29 (0.51)	1.17 (0.53)	1.22 (0.47)	1.30 (0.47)	1.26 (0.29)	1.52 (0.68)	0.0008
fasting insulinaemia	pmol.1^-1^	114 (72)	77 (48)	103 (78)	126 (80)	127 (69)	141 (69)	<0.0001
metabolic syndrome	%	86	59	82	94	94	100	<0.0001
metabolic syndrome score	0/5 to 5/5	3.7 (1.1)	2.6 (1.0)	3.3 (0.9)	3.8 (0.8)	4.3 (0.9)	4.7 (0.6)	<0.0001
liver steatosis	%	64	27	55	76	80	83	<0.0001

Results are expressed as means (1 SD) or proportions (%). HOMA S and HOMA B: insulin sensitivity and β-cell function determined from Homeostatic Model Assessment. Q: quintile.

As regards glucose-lowering therapies, 58% of patients were treated with metformin; 41% with a β-cell stimulant; and/or 50% with exogenous insulin. There was a significant trend for higher insulin use or higher dosage across quintiles: 35% and 0.42 UI.day^-1^.kg^-1^ (Q I) to 58% and 1.13 UI.day^-1^.kg^-1^ (Q V; *p* 0.0058 and <0.0001, respectively). LLD(s) were given to 63% of the whole cohort, with statin; fenofibrate and/or ezetimibe prescribed to 51%; 19% and/or 3%. There was a significant trend for increased fenofibrate use across Qs: 9% (Q I) to 39% (Q V; *p* <0.0001). None of the patients were treated with niacin.

Mean baseline TG values were 206 (191) mg.dL^-1^ in the entire cohort. There were no significant differences across Qs in baseline LDL-C and non-HDL-C. As for current lipid values, mean LDL-C was 100 (36) mg.dL^-1^; HDL-C 52 (15) mg.dL^-1^; and TG 160 (105) mg.dL^-1^. Mean non-HDL-C was 132 (42) mg.dL^-1^. There were significant trends for increasing values across Qs in non-HDL-C, apoB_100_, and apoB_100_.apoA1^-1^ (*p* 0.0078, <0.0001 and <0.0001, respectively). There were also significant trends for decreasing values across Qs in apoA1 and in LDL size’s surrogate [LDL-C/apoB_100_] (both *p* <0.0001). Increasing levels across Qs were also observed for _hs_CRP, fibrinogen, Big ET-1, uric acid, cystatin C, and magnesium, and for decreasing levels in SHBG (Table 3). In the entire cohort, mean eGFR was 76 (28) ml.min^-1^.1.73m^-2^, and albuminuria 85 (273) μg.mg creatinine^-1^, without significant differences between Qs.

**Table 3:** Laboratory values

		**all patients**	**Q I**	**Q II**	**Q III**	**Q IV**	**Q V**	**P**
n		340	68	68	68	68	68	
atherogenic dyslipidemia	%	35	1	12	25	56	82	~
pre-LLD lipids								
LDL-C	mg.dl^-1^	150 (35)	149 (25)	159 (46)	155 (28)	152 (39)	137 (28)	NS
non-HDL-C	mg.dl^-1^	187 (41)	172 (28)	191 (54)	193 (36)	193 (46)	187 (36)	NS
HDL-C	mg.dl^-1^	52 (15)	69 (17)	54 (8)	50 (9)	46 (11)	42 (8)	~
triglycerides	mg.dl^-1^	206 (191)	120 (66)	159 (80)	205 (98)	225 (204)	317 (308)	~
current lipids								
total cholesterol	mg.dl^-1^	185 (43)	195 (35)	185 (45)	182 (43)	181 (41)	180 (51)	NS
LDL-C	mg.dl^-1^	100 (36)	102 (31)	101 (38)	104 (38)	102 (34)	90 (36)	NS
non-HDL-C	mg.dl^-1^	132 (42)	121 (33)	126 (42)	132 (41)	137 (39)	145 (49)	0.0078
apoB_100_	mg.dl^-1^	92 (27)	80 (22)	90 (26)	91 (25)	96 (26)	103 (30)	<0.0001
HDL-C	mg.dl^-1^	52 (15)	74 (11)	59 (6)	50 (4)	44 (4)	35 (5)	~
apoA1	mg.dl^-1^	164 (30)	195 (24)	164 (20)	166 (23)	152 (22)	143 (32)	<0.0001
triglycerides	mg.dl^-1^	160 (105)	91 (42)	121 (55)	142 (49)	176 (73)	272 (152)	~
apoB_100_.apoA1^-1^		0.54 (0.19)	0.39 (0.12)	0.52 (0.16)	0.52 (0.14)	0.62 (0.17)	0.67 (0.22)	<0.0001
LDL-C.apoB^-1^		1.05 (0.30)	1.23 (0.27)	1.07 (0.30)	1.08 (0.26)	1.00 (0.26)	0.86 (0.25)	<0.0001
_hs_CRP	mg.dl^-1^	0.50 (0.68)	0.28 (0.38)	0.46 (0.90)	0.53 (0.69)	0.50 (0.48)	0.70 (0.74)	0.0082
fibrinogen	mg.dl^-1^	340 (78)	313 (66)	340 (76)	334 (72)	348 (69)	365 (97)	0.0025
Big ET-1	pg.ml^-1^	6.29 (2.31)	5.55 (2.45)	5.14 (1.21)	6.31 (2.53)	6.71 (1.84)	7.88 (2.46)	<0.0001
uric acid	mg.dl^-1^	5.3 (1.7)	4.8 (1.6)	4.8 (1.5)	5.6 (1.4)	5.4 (1.7)	5.8 (1.8)	0.0002
cystatin C	mg.1^-1^	0.88 (0.30)	0.75 (0.23)	0.78 (0.28)	0.88 (0.26)	0.91 (0.28)	1.03 (0.38)	<0.0001
magnesium	mEq.dl^-1^	1.63 (0.20)	1.67 (0.22)	1.71 (0.16)	1.61 (0.17)	1.64 (0.21)	1.56 (0.19)	<0.0001
SHBG	nmol.1^-1^	48 (33)	66 (45)	45 (28)	43 (23)	46 (29)	40 (30)	<0.0001

Results are expressed as means (1SD) or proportions (%). apo: apoliprotein; C: cholesterol; ET: endothelin; HDL: high-density lipoprotein; hsCRP: high sensitivity C-reactive protein; LDL: low-density lipoprotein; LLD: lipid-lowering drug(s); Q: quintile; SHBG: sex hormone-binding globulin; NS: not significant.

**Figure 1:**
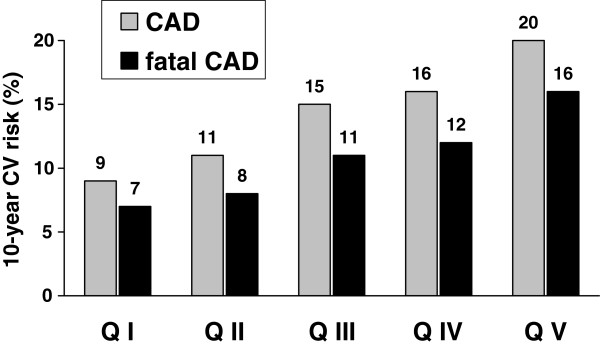
**United Kingdom Prospective Diabetes Study Risk Engine’s 10-year absolute predicted risk of developing non-fatal or fatal coronary artery disease (CAD; grey bars) or fatal CAD (solid bars) in 258 T2DM females according to quintiles of log (TG)/HDL-C ranking distribution.** Within each quintile, data were obtained from subsets of patients (in % for each quintile) in primary cardiovascular prevention (Q I: 81%; Q II: 81%; Q III: 69%; Q IV: 78%; and Q V: 71%). HDL-C: high-density lipoprotein cholesterol; TG: triglycerides. Significance of differences between quintiles: both P<0.0001 (CAD and fatal CAD).

In the entire cohort, 258 patients were in primary CV prevention, and as a result eligible for UKPDS risk estimation. Their mean 10-year absolute CV risk prediction was: 13 (11) % (*CAD*); 10 (10) % (*fatal CAD*); 11 (16) % (*stroke*) and 2 (3) % (*fatal stroke*). Figure 1 illustrates UKPDS risk for CAD, after adjustment for inter-quintile differences in mean age: there were significant trends for stepwise heightened risk across Qs: 9% (CAD) and 7% (fatal CAD) (Q I) to 20% (CAD) and 16% (fatal CAD) (Q V; both *p* <0.0001). No increasing trends across Qs were observed for risk of stroke or lethal stroke (*not illustrated*).

## Discussion

The main findings of the present study are fourfold. Firstly, we observed a high prevalence of AD in T2DM females, with 35% affected, similar to that observed in T2DM males. Secondly, the AD ratio *log*(TG)/HDL-C is an easy means to estimate IR, and was strongly related to a cardiometabolic phenotype characterized by IR, MetS, hyperinsulinemia, central fat accretion, sarcopenia, liver steatosis and sedentary lifestyle. Thirdly, this ratio was linked to a stepwise gradient for residual risk of future CAD. Lastly, this ratio was related to decreased insulin secretion, β-cell function loss, poorer glycemic control and higher frequency of microangiopathies. The AD ratio may therefore allow to easily identifying a comprehensive modifiable component of non-LDL, lipid-related residual vascular risk, as well as pinpoint worsening in glucose homeostasis determinants [5-7].

The hallmark of AD is decreased HDL-C level together with raised TG, with LDL-C often only marginally elevated [2-5,33-40]. Computing a ratio from fasting TG (*numerator*) and fasting HDL-C (*denominator*) allows not only for grading AD severity as a continuous variable, but also for incorporating the effect of mutually-reinforcing or diverging confounders affecting both fraction’s components and that of one variable on the variance of the other [7]. An analysis of isolated TG or HDL-C, both indispensable determinants to define AD, as standard continuous variables would not be able to incorporate a measure of AD. Using consensual MetS cutoffs for defining decreased HDL-C and elevated TG, we observed a high prevalence of AD in T2DM females (more than one-third). In comparison with *log*(TG)/HDL-C values from non-diabetic female controls, the mean AD ratio in T2DM females was well above normal values, from the fourth AD quintile onwards. This demonstrates that >40% of this population may present with AD of varying severity. This finding was expected, as raised TG and low HDL-C levels are used to define MetS phenotype, and as the latter is highly prevalent (86%) in T2DM [17,18].

To rule out any confounding effect of LLD, we used *baseline* TG and HDL-C to compute the AD ratio, prior to anti-dyslipidemic drug(s). This proved appropriate, as the mean difference between baseline and current TG levels in this study averaged 46 mg.dL^-1^. This approach allowed thus to precisely establishing the true, underlying magnitude and frequency of AD in T2DM females [5,7]. There is a known gender difference in HDL-C and TG levels in the general population, also observed in this diabetic sample, with T2DM females exhibiting higher mean HDL-C and lower TG values than their T2DM males, by an average 6 and 12 mg/dl, respectively (*data not shown*) [12-14]. As for lifestyle-related confounders of AD prevalence/severity, our data showed a significant decreasing trend across AD quintiles for alcohol intake, the inverse association being expected due to ethanol’s HDL-C-raising effects. On the other hand, we observed a trend for lower educational level. This may promote less healthy behaviours, and result in adverse (i.e. less cardioprotective) anthropometrics and body composition.

The AD ratio was associated, in a stepwise gradation, with higher levels of numerous non-lipid cardiometabolic markers: fasting insulinemia; _hs_CRP; fibrinogen; Big ET-1; uric acid, and cystatin C. Compensatory hyperinsulinemia is likely to represent a major underlying driver for both AD and for the proatherogenic changes observed in non-LDL lipids and non-lipid CV markers. Endothelin-1 upregulation and/or nitric oxide release imbalance by endothelial cells is another putative pathway linking IR to macroangiopathy. Thus, insulin stimulates endothelin-1 production and action through MAP-kinase-dependent pathways, while ET-1 induces IR and reactive hyperinsulinemia. Such a feed-back loop may contribute to the pathogenesis of macrovascular disease in cardiometabolic states [32]. Expectedly, SHBG levels significantly decreased across quintiles, since IR/portal hyperinsulinemia are associated with decreased liver production and lower levels of this androgen transporter [4,5,24,25,32,39].

Accumulating evidence links AD in T2DM not only to residual macrovascular risk, but also to residual risk for new-onset or progression of microangiopathy [11]. In this study, *log*(TG)/HDL-C was associated with lesser residual insulin secretion, as well as with more severe hyperbolic product (BxS) loss over time, and heralded poorer glycemic control and earlier or more intensive need for stepping-up glucose-lowering therapies, as we previously reported in T2DM males [7,22,23,29,41,42]. The AD ratio was associated with predicted CAD risk and prevalent overall microangiopathy. When patients were analyzed according to quitiles of non-HDL-C, another variable which rises alongside the severity of AD, we found no association between the latter and the frequency of microangiopathy. This, together with the independence from diabetes control comforts the relevance of resorting to the *log*(TG)/HDL-C ratio to estimate a modifiable lipid-related component of residual vascular risk linked to AD. Noteworthy, treatment with fenofibrate, a PPAR-α agonist that specifically targets AD, exerts beneficial effects on macrovascular disease in T2DM patients with AD, but also confers vascular protection against retinal, renal and lower-limb microangiopathies [3,4,9-11].

The present study has several limitations. Firstly, as a cross-sectional hypothesis-generating study, it does not allow for inferring causality relationships. Secondly, as we used routine measurement of HDL-C, without HDL subclass assessment, we cannot discriminate between atheroprotective *vs.* dysfunctional, less atheroprotective or even atherogenic particles. As the latter often concur with decreased HDL-C, a low HDL-C level in the ratio may nevertheless capture to some extent these qualitative defects. Finally, the population under study was mostly of European Caucasian ancestry, and the present findings require replication in other racial/ethnic subgroups. Moreover, the observed associations were documented from females patients with established T2DM, and may not necessarily exist in newly-diagnosed T2DM females, nor in other types of diabetes or in women with normal glucose homeostasis [43-45].

In conclusion, *log*(TG)/HDL-C allows for grading AD and estimating non-LDL-related residual vascular risk in T2DM females. This AD ratio negatively associates with the two major determinants of glucose homeostasis. This means that patients with more severe AD will have poorer metabolic control in addition to higher predicted CAD risk. The ratio *log*(TG)/HDL-C appears a simple, cheap noninvasive means to better precise gender-specific assessment and care of the female patient with diabetes. Identifying patients with abnormal AD ratio should allow improving selection and follow-up of those most at risk, more likely to benefit from available or newer therapies targeting hypertriglyceridemia and/or hypo-HDL-cholesterolemia.

## Competing interests

The authors declare that they have no competing interests.

## Authors’ contributions

MPH was involved in the design and conduct of the study, as well as in collecting patients’ data; MPH, SAA and MFR were involved in analysis and interpretation; preparation and review of the manuscript, and decision to submit the manuscript for publication. All authors read and approved the final manuscript.
